# CircRNA circ_0023984 promotes the progression of esophageal squamous cell carcinoma via regulating miR-134-5p/cystatin-s axis

**DOI:** 10.1080/21655979.2022.2063562

**Published:** 2022-04-20

**Authors:** Ge Yang, Yu Zhang, Hongni Lin, Jinnbo Liu, Shengjie Huang, Wei Zhong, Chao Peng, Lin Du

**Affiliations:** a Department of Clinical Laboratory, Affiliated Neijiang Second People’s Hospital of Southwest Medical University, Neijiang, P.R, China; b Department of Clinical Laboratory, The First Affiliated Hospital of Southwest Medical University, China Neijiang; c Scientific research department, Sichuan Neijiang Health Vocational College, China Neijiang; d Nuclear medicine department, Affiliated Neijiang Second People’s Hospital of Southwest Medical University, Neijiang, P.R, China; e Department of intestine surgery, Affiliated Neijiang Second People’s Hospital of Southwest Medical University, Neijiang, P.R, China

**Keywords:** Circ_0023984, miR-134-5p, CST4, esophageal squamous cell carcinoma, treatment targets

## Abstract

Recent studies have shown that circRNAs can act as oncogenic factors or tumor suppressors by sponging microRNAs (miRNAs). The upregulation of circ_0023984 was reported in esophageal squamous cell carcinoma (ESCC). However, its functional role in ESCC remain unclear. In the present study, circ_0023984 expression in ESCC cells and tissues were analyzed by quantitative real-time polymerase chain reaction (qRT-PCR) and Western blotting (WB). Subcellular fraction experiment was performed to determine relative nuclear-cytoplasmic localization. The loss-of-function effects of circ_0023984 in ESCC cell lines were investigated by shRNA-mediated knockdown. Functional assays including cell Counting Kit-8 (CCK-8), 5-Ethynyl-2’-deoxyuridine (EDU) incorporation, colony formation and Transwell migration assays were conducted to assess the malignant phenotype. The interaction between the two molecules was analyzed by RNA pull-down, luciferase reporter assay and RNA immunoprecipitation (RIP). The subcutaneous tumor model in nude mice was used to assess the role of circ-0023984 in tumorigenesis. We found that ESCC patients with high circ_0023984 expression was associated with a poor prognosis. The knockdown of circ_0023984 suppressed cell growth, invasion, and migration in ESCC cells. Circ_0023984 interacted with miR-134-5p and inhibited its activity, which promoted the expression of CST4 (Cystatin-S). Circ_0023984 also regulated tumorigenesis in a CST4-dependent manner. Together, our study indicates that the oncogenic role of Circ_0023984 is mediated by miR-134-5p/CST4 Axis in ESCC, which could serve as potential targets for future therapeutic strategies.

## Highlights

- Circ_0023984 is upregulated in ESCC tissues and cell lines.
- Circ_0023984 silencing suppresses the malignant phenotypes in ESCC cells.
- Circ_0023984 maintains CST4 expression via sponging miR-134-5p.

## Introduction

Esophageal cancer (EC) is a malignancy with high incidence and high mortality [1]. EC is ranked as the eighth cause of cancer-related morbidity worldwide, while its fatality ranks the sixth place among malignancies [2]. Esophageal squamous cell carcinoma (ESCC) is the most prevalent oral cancer in China, accounting for more than 90% of all pathological types of EC [3]. ESCC is characterized of high aggressiveness and poor prognosis [4]. Despite of extensive research efforts into the pathogenesis and treatment of ESCC, the mechanisms underlying its progression are still perplexing.

CircRNAs are noncoding RNAs (ncRNAs) with a closed-loop structure in which the back splicing joins the 3’ and 5’ end of a RNA molecule [5]. Compared with the linear counterparts, cicrRNAs show a relatively high stability and resistance to ribonuclease digestion [6,7]. They are endogenous single-stranded RNAs primarily produced from pre-mRNAs and expressed in a in tissue- and development-specific pattern [8]. MicroRNAs (miRNAs) are another class of ncRNAs with 20–22 nucleotides in length. The dysregulation of miRNAs has been implicated in the regulation of cell growth, development, metabolism, differentiation, cell migration and apoptosis [9]. miRNAs usually interact with the 3’- or 5’-untranslated region (UTR) of target mRNA to disrupt mRNA stability or induce translation repression, which adversely modulate target gene expression. CircRNAs add another layer of gene expression control by modulating the availability of microRNAs. The sponging effect of circRNAs has been recognized as a general mechanism for circRNAs to adsorb miRNA targets and undermine their interactions with target mRNAs [10,11]. The mature miRNAs are produced from endogenous pri-miRMA in the nucleus, which are processed to short pieces of miRNAs by a series of nucleases [12]. After maturation, miRNAs are integrated into the RNA-induced silencing complex (RISC) by interacting with Argonaute (AGOs) proteins, and regulate the stability or translation of target mRNAs through RISC in a sequence-dependent manner [13].

The deregulation of circRNAs have been widely reported in tumor biology [14]. The upregulation or downregulation circRNAs have been found to contribute to cancer genesis and development [15]. For example, in hepatocellular carcinoma, circRNA-5692 enhances DAB2IP (DAB2 interacting protein) expression through sponging miR-328-5p, thereby inhibiting the development of hepatocellular carcinoma [14]. In gastric cancer, circ-SERPINE2 sponges miR-375 and regulates YWHAZ (tyrosine 3-monooxygenase/tryptophan 5-monooxygenase activation protein zeta) expression to promote the development of gastric cancer [15]. In ESCC, circ_0006948 has been showed to sponge different miRNAs to regulate the tumor progression [15]. As described in a previous study, circ_0023984 can promote ESCC progression by regulating miR-433-3p/REV3L (REV3 Like, DNA-directed polymerase zeta catalytic subunit) axis [16]. Since there are multiple binding sites for miRNAs in a circRNA, delineating the full picture of downstream miRNA targets of circRNAs can enrich the understanding of the regulatory network in cancer progression. Indeed, circ_0023984 was reported to be highly upregulated in ESCC samples by another previous study [17], indicating its oncogenic activity in ESCC cells.

In this study, we hypothesized that the upregulation of circ-0023984 in ESCC contributes to the malignant progression by sponging downstream miRNA target. We first validated its upregulation in ESCC tissues and cells, as well as its oncogenic role. We further identified a binding site for miR-134-5p on circ_0023984, and a binding site of miR-134-5p in the 3ʹUTR of CST4. We therefore reasoned that circ_0023984 may regulate miR-134-5p/CST4 axis to mediate the progression of ESCC. Through a series of functional experiments, our study revealed that the oncogenic role of Circ_0023984 is dependent on the modulation of miR-134-5p/CST4 Axis in ESCC, which could serve as potential targets for future therapeutic strategies.

## Materials and methods

### Bioinformatics analysis

This study retrieved the data of circRNA expression profile of ESCC tissues from the dataset GSE131969 in GEO database, which includes 3 ESCC tissue samples and 3 para-cancerous normal tissue samples [17]. The mRNA expression profiles of ESCC patients were obtained from the TCGA data using Starbase online tool (http://starbase.sysu.edu.cn/), which contains a total number of 162 ESCC samples and 11 para-cancerous normal tissues.

### Patients and samples

This study collected ESCC tissues and paired paraneoplastic tissues from 70 ESCC patients at the Affiliated Neijiang Second People’s Hospital of Southwest Medical University. All patients had a clear clinicopathological diagnosis. Fresh tissue samples were processed within 10 min after surgical resection, and the para-cancerous tissues were confirmed to be free of tumor cell infiltration by pathologic tissue testing. All the tissues were rapidly frozen within liquid nitrogen, followed by long-term preservation at −80°C. This study was approval from the Ethics Committee of Affiliated Neijiang Second People’s Hospital of Southwest Medical University. All the patients signed the informed consent. Inclusion criteria: patients had not undergone radiotherapy before surgery; with complete follow-up data of three years; and the location of the tumor was located in the thoracic segment of the esophagus.

### Cell culture

Cell lines were acquired from Shanghai Cell Bank, Chinese Academy of Sciences (Shanghai, China), which includes ESCC cell lines (EC109, EC9706, KYSE30, KYSEE150, KYSE410) and normal esophageal epithelial cell line (SHEE). The cells were cultivated in DMEM with 1% L-glutamine, 1% penicillin-streptomycin as well as 10% fetal bovine serum (FBS, GiBCO, CA, USA) under 5% CO2 condition.

## Lentivirus infection for stable circ_0023984 knockdown

Lentivirus containing negative control (NC) and circ_0023984 short hairpin RNA (shRNA) (sh-NC and sh-circ_0023984 #1, #2, #3) was purchased from GenePharma Co., Ltd. (Shanghai, China). Cells at logarithmic growth period were seeded (5 × 10^4^/well) into the 24-well plates and cultured overnight. On day 2, the culture medium was replaced with fresh medium containing 6–8 μg/ml Polybrene (Sigma-Aldrich GmbH, Sternheim, Germany), and KYSE150 and KYSE30 cells were transfected with 0.5 mL lentivirus for 48 h. Afterward, the culture medium was replaced with medium containing 2 µg/ml of puromycin (Merck, London, UK) to select for the cell infected with the lentivirus for 2 weeks. After selection, the circ_0023984 expression was examined by qRT-PCR to verify the stable knockdown [18].

## Cell transfection

CST4 overexpressing plasmid (with neomycin resistance gene selection marker), miR-134-5p mimic and miR-134-5p inhibitor and their negative control (NC) were purchased from HanBIO (Shanghai, China). Cell transfection was performed using Lipofectamine® 3000 reagent (Invitrogen, CA, USA). In 6 well plate, 60% confluent cells were transfected with 100 nM of microRNA mimic or inhibitor or 6 ug of CST expression plasmid according to manufacturer’s instruction [19]. For stable CST4 expression, 48 h after transfection the cells were selected with 500ug/ml G418 for 2 weeks.

## Cell counting kit-8 (CCK-8) assay

Cells at logarithmic growth period were harvested and inoculated (5 × 10^4^/well, 100 μl) in 96-well plates and cultured for 0, 24, 48, and 72 hours, respectively. Subsequently, 10-μL CCK8 reaction solution (5 mg/ml, Shanghai, Beyotime) was added to the cell culture at indicated time point and incubated for 3 h in a humidified cell culture incubator. The light absorption value (OD value) in each condition was captured at 450 nm wavelength on microplate reader (Biorad, USA) [18].

## 5-Ethynyl-2’-deoxyuridine (EDU) incorporation assay

Cells (2 × 10^5^/well) were inoculated in the 24-well plates, and EDU staining kit (Click-iT® EdU, Invitrogen) was used for Edu incorporation assay. When cells reached 80%, the culture medium was replaced with the medium containing 1x EdU and incubated for 2 h. After rinsing by PBS, 4% formaldehyde was added to fix cells for a 15 min, followed by 20 min incubation with 0.5% Triton X-100 in PBS. Then, Click-iT reaction mixture (0.5 mL) added to the fixed cells for 30-min incubation. The stained cells were washed by PBS with 3% BSA, and counterstained with DAPI (1 g/mL) for 15 min in the dark environment. After staining and wash with PBS, the images were captured under Leica AM6000 microscope [20].

## Colony formation assays

Cells at logarithmic growth period were seeded in 12-well plates at a density of 5 × 10^3^ cells per well and incubated for two weeks. Afterward, cells were rinsed with PBS twice, and fixed by 4% paraformaldehyde for 20 min. After wash with PBS, the cells were stained with 0.4% crystal violet staining (Sigma) for 5 mins. After discarding crystal violet staining solution, cells were washed with distilled water. The colony number in each well was determined using an inverted microscope at × 40 magnification (Zeiss, Oberkochen, Germany) [21].

## Transwell migration and invasion assay

Cell invasion and migration were analyzed by 24-well Transwell chambers containing polycarbonate membrane (pore size, 8 μm; Merck Millipore Bioscience, Germany). In the invasion assay, 5 × 10^4^ cells in 200 µl serum-free medium were seeded into Matrigel-coated upper chamber (diluted with serum-free DMEM at 1:10; BD Biosciences, Belgium); for the migration ability assay, no Matrigel coating was performed. 600 μl DMEM medium containing 20% FBS was added into bottom chamber for 12-h incubation. At the end of the experiment, Transwell chambers were gently removed with forceps, and a sterile cotton was utilized to wipe the upper layer of unpenetrated cells. Cells on the membrane were fixed using 4% paraformaldehyde for 10 min, and then stained with 1% crystal violet (Sigma) for 5 min. The cell images were captured using an inverted microscope at × 200 magnification (Zeiss, Oberkochen, Germany) in three randomly selected fields in the middle of the membrane. Image J software (National Institute of Health, Bethesda, Maryland, USA) was utilized to count and quantify cell number [18].

## Subcellular fraction assay

Cells at logarithmic growth period were harvested. After digestion with 0.25% trypsin, 1 × 10^7^ cells were collected, lysed, and centrifuged according to the instructions of PARIS™ Kit (Ambion, Austin, TX). The cytoplasmic fraction in the supernatant was collected, and the precipitated nuclear fraction lysed by nucleus lysis buffer. The collected cytoplasmic fractions and nuclear lysates were mixed with 2× lysis binding solution, respectively, then passed through a filter cartridge for total RNA extraction [22]. The circ_0023984 level in the nuclear and cytoplasmic fractions were measured by qRT-PCR. GAPDH and U6 served as positive controls for nuclear and cytoplasmic fractions.

## RNaseR digestion assay

Total RNA extract (5 μg) from KYSE30 and KYSE150 cells were collected by Trizol (Invitrogen, Carlsbad, CA, USA). The RNA sample was divided equally into two portions: one was used for RNase R digestion (RNase R+ group), and the other was used as control (RNase R- group). The two portions of samples were incubated at 37°C for 25 min. circRNA_0023984 and NOX4 mRNA level in each sample was measured through RT-qPCR [23].

## Target prediction and dual luciferase reporter assay

The circBank web server (http://www.circbank.cn/) was utilized to perform circRNA target prediction [24], and the Targetscan web server (http://www.targetscan.org/vert_71/) was employed for miRNA target prediction [25]. 1.5 μg Luciferase reporter vector containing WT binding site (pGL3-circRNA_0023984-WT, pGL3-CST4-WT) or mutated binding site (pGL3-circRNA_0023984-MUT, pGL3-CST4-MUT) were transfected into KYSE30 and KYSE150 cells (1 × 10^5^/well) in 12-well plates, in the presence of 100 nmol/L miR-134-5p mimic or NC mimic and 1 µg of internal reference plasmid expressing renilla luciferase plasmid for 48 h. Afterward, renilla and firefly luciferase activities were analyzed on a luminescence plate reader [14]. The relative firefly luciferase activity in the reporter plasmid was normalized to that of renilla luciferase control plasmid.

## RNA pull-down assay

The total cell lysates isolated from KYSE150 and KYSE30 cells were incubated with 100 ng biotin-labeled scramble control oligos or circRNA_0023984 probe for 2h. 10% of the lysates was saved as the input. Then 100 µl streptavidin-coated magnetic beads (Invitrogen, CA, USA) were mixed with the solution for 4 h incubation. A magnetic bar was used to pull down the magnetic beads and associated nucleic acids, then the samples were washed 4 times with high salt wash buffer. Both the input and the elutes from the pull-down were purified with Trizol reagent and quantified by RT-qPCR [26].

## RNA binding protein immunoprecipitation (RIP) assay

EZ-MagnaRIP Kit (Millipore, MA, USA) was utilized to conduct RIP assay. Cells were lysed using IP lysis buffer and incubated with Pierce™ Protein A/G Magnetic Beads (Thermo Fisher Scientific, USA) conjugated with a rabbit anti-Ago2 (Abcam, ab32381) antibody or with a negative control normal rabbit anti-IgG (Abcam, ab188776). The mixture was incubated at 4°Cfor 4 h. Magnetic beads were precipitated using a magnetic bar and the precipitated samples were washed three times with lysis buffer. The eluted samples were purified with Trizol reagent and quantified by RT-qPCR analysis [27].

## Quantitative real-time polymerase chain reaction (RT-1PCR)

Cells cultured under logarithmic growth phase were subject to different treatments and Trizol reagent (Invitrogen, Carlsbad, CA, USA) was used for total RNA extraction. miR-134-5p and circ_0023984 expression was measured using TaKaRa one-step RNA PCR kit (TaKaRa Bio Inc, Japan). The following qPCR conditions were used: 95°C, 5 min; 40 cycles of 95°C, 5 min; 95°C, 10s; 60°C, 45s. GAPDH and U6 served as the endogenous controls for circRNA and miRNA, respectively. 2–∆∆Ct method was used to analyze the relative expression level [27]. The primer sequences used in the study were listed below:

circ_0023984: 5´-TCCGGAGCAATAAGCCAGTC-3´(forward); 5´-TTAAGACTGATGCAGCCGGG-3´(reverse).

miR-134-5p: 5´-GCAGTGTGACTGGTTGAC-3´(forward); 5´-CAGTGCGTGTCGTGGAGT -3´ (reverse).

U6:5´-CTCGCTTCGGCAGCACA-3´(forward); 5´-AACGCTTCACGAATTTGCGT-3´ (reverse).

CST4: 5´-CCTCTGTGTACCCTGCTACTC-3´(forward);

5´-CTTCGGTGGCCTTGTTGTACT-3´ (reverse).

GAPDH: 5´-TCAAGAAGGTGGTGAAGCAGG-3´ (forward);

5´-TCAAAGGTGGAGGAGTGGGT-3´ (reverse).

## Western-blotting (WB) assay

RIPA (Beyotime Biotechnology, Shanghai, China) was used for extracting total protein from cells. The protein concentration was determined by BCA assay (Beyotime Biotechnology, Shanghai, China). Protein samples (50 μg) were separated by SDS-PAGE and transferred on PVDF membrane (Millipore Burlington, MA, USA). The membranes were blocked with 5% skimmed milk in Tris Buffered saline Tween (TBST) buffer, followed by incubation with anti-CST4 (Abcam, Cambridge, UK) or anti-Actin mouse monoclonal (Abcam, Cambridge, UK) antibody overnight at the temperature of 4°C. The membranes were washed with TBST buffer twice and further incubated with HRP-conjugated secondary antibody (Abcam, Cambridge, UK) for 1 h. The ECL reagent (Santa Cruz, TX, USA) was used to visualize proteins bands under Bio-rad chemiluminescence detector (Bio-Rad, Hercules, CA). Quantity One software (Bio-Rad, Hercules, CA) was employed for densitometry analysis of protein bands [27].

## *In vivo* tumorigenesis in nude mice

All animal procedures were approved by the Ethics Committee of Affiliated Neijiang Second People’s Hospital of Southwest Medical University. Eighteen male immunodeficient nude mice weighing 30–40 g were randomly divided into three groups (6 mice in each group) [1]: sh-NC group (injected with KYSE150 cells infected with sh-NC) [2], sh-circ_0023984 (injected with KYSE150 cells infected with sh-circ_0023984) [3]. sh-circ_0023984+ CST4 (injected with KYSE150 cells infected with sh- circ_0023984 and stably transfected with CST4 expression vector). 0.2 mL of cell suspension containing 1 × 10^7^ cells was injected into the flank of each mice. Tumor volume were monitored 7, 14, 21, 28 and 35-days post-injection, respectively. Tumor length and short diameter of each group of nude mice were measured and recorded with a Vernier caliper after tumor appearance. Tumor volume calculation formula: V = 0.5× long diameter × short diameter^2^. Seven weeks after inoculation, all the mice were euthanized by CO2 asphyxiation and followed by cervical dislocation. The tumors of terminally dead mice were resected for weight measurement and further analysis.

## Immunohistochemistry (IHC)

4% paraformaldehyde solution was used to fix tumor tissues under 4°C for 48 h. After that, tissues were cut into small pieces and embedded in paraffin, and the cut into 4-μm thick slices using microtome. Tissue section was dehydrated and antigen unmasking was performed by heating the section in a microwave submersed in 1X citrate unmasking solution. After three times washes in TBST buffer for 5 min, the section was blocked for 1 hour in TBST buffer with 5% normal Goat Serum, and then incubated with primary antibody: Ki-67 (Abcam, Cambridge, UK) and CST4 (Abcam, Cambridge, UK) under 4°C for overnight. The section was stained with HRP-conjugated secondary antibody for a 30-min period under ambient temperature, and color development was achieved by DAB staining for 10 min. Hematoxylin was used to nucleus counterstain and the images were taken using a microscopy (Olympus, BH-40, Tokyo, Japan) [18].

## Statistical analysis

SPSS21.0 (SPSS-Science, Chicago, IL) was used for all statistical analyses. The test of normality is the premise of all data analysis. Measurement data conforming to normal distribution were presented in a form of mean ± SD. The statistical difference between two groups was analyzed using unpaired student’s t tests. Comparisons of multiple groups were conducted using one-way analysis of variance (ANOVA) with Tukey’s post hoc test for pairwise comparison. A difference of *P* < 0.05 was considered as statistical significance.

## Results

In this study, we found that circ_0023984 was highly expressed in ESCC tissues and cell lines, which was associated with poor overall survival in 70 ESCC patients. Circ_0023984 knockdown significantly decreased cell proliferation, colony formation and migration ability. We further showed that circ_0023984 could interact with miR-134-5p to regulate CST4 expression. Circ_0023984 sponged miR-134-5p and released the inhibition on CST4 expression in ESCC cells. The involvement of CST3 and circ_23984 in ESCC tumorigenesis was also evaluated in mouse model. The silencing of circ_0023984 impaired xenograft growth, and CST4 overexpression partially rescued tumorigenesis upon circ_0023984 silencing.

## Circ_0023984 is upregulated in ESCC tissue and cell line

**Figure 1.**
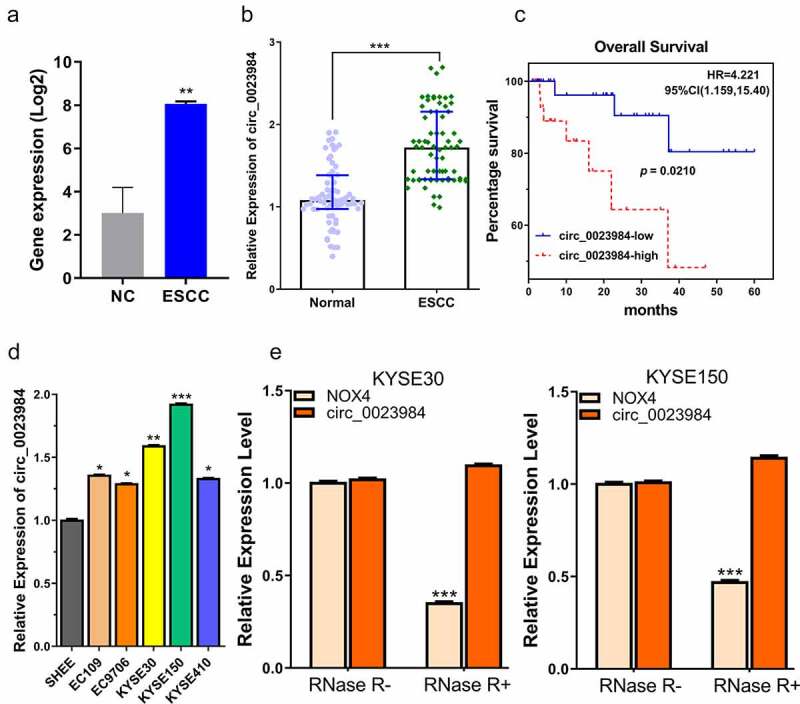
Circ_0023984 shows high expression in ESCC tumor and cell lines.A, circ_0023984 is upregulated in ESCC in GSE131969 dataset; NC (normal tissues), ESCC (Esophageal Squamous Cell Carcinoma), data are expressed in Log2 FPKM. B, circ_0023984 level measured by RT-qPCR in ESCC tumor tissues (ESCC) and para-cancerous tissues (NC) from 70 ESCC patients; C, K-M survival analysis for assessing the overall survival in 70 patients; D. RT-qPCR analysis of circ_0023984 expression in ESCC cell lines (EC109, EC9706, KYSE30, KYSEE150, KYSE410) and normal esophageal epithelial cell line (SHEE); E, RT-qPCR analysis of NOX4 mRNA and circ_0023984 level after RNase R digestion. Statistics: A. E: students’ t test; B: Mann–Whitney U test; C: log-rank test; D: one-way analysis of variance (ANOVA) with Tukey’s post hoc test. * P < 0.05; **P < 0.01; ***P < 0.001.

We first analyzed the previously published dataset GSE131969, which includes 3 ESCC tissue samples and 3 para-cancerous normal tissue samples [17]. In the dataset the expression of circ_0023984 is significantly higher in ESCC tumor samples (Figure 1a), indicating a potential contribution to the progression of ESCC. To further confirm the expression of circ_0023984 in ESCC, we collected ESCC tissues and paired paraneoplastic tissues from 70 ESCC patients and examined circ_23984 by RT-qPCR. Consistently, circ_0023984 was significantly upregulated in ESCC tumor samples (Figure 1b). In order to demonstrate the potential role of circ_0023984 in ESCC prognosis, 70 ESCC patients were divided into circ_0023984-low and -high expression group (n = 35 in each group) based on the median value of circ_0023984 expression. Survival analysis by Kaplan-Meier curve showed that the overall survival in the high expression group was significantly poorer than the low expression group (Figure 1c, p<0.05). We also analyzed the expression of circ_0023984 in esophageal epithelial SHEE cells and ESCC cell lines (including KYSE30, KYSEE150, KYSE410, EC109, EC9706) by RT-1PCR. As a result, circ_0023984 levels were elevated in multiple ESCC cell lines (Figure 1d), and KYSE30 and KYSE150 cells showed a relatively higher level of expression.

To confirm the circular structure of circ_0023984, we performed RNase R digestion to of the RNA samples isolated from KYSE30 and KYSE150 cells, and examined the change in abundance of linear NOX4 mRNA (the gene locus where circ_0023984 derives) and circ_0023984. NOX4 mRNA level was remarkably reduced after RNaseR treatment, while there was no significant change of circ_0023984 level (Figure 1e), which suggests the closed-loop structure of circ_0023984 and its resistance to RNase R digestion.

## The knockdown of circ_0023984 suppresses ESCC cell growth, invasion and migration

**Figure 2.**
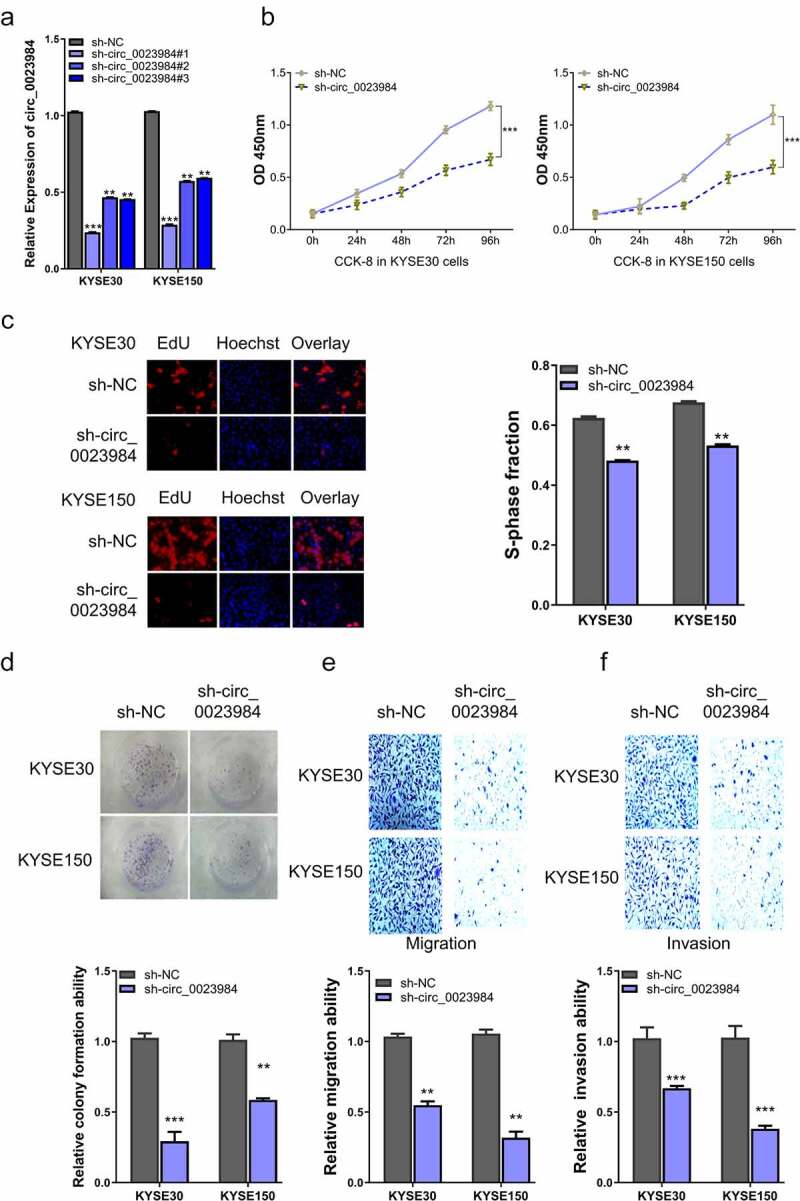
Knockdown of circ_0023984 suppresses ESCC cell growth, invasion and migration.A, RT-qPCR analysis of the knockdown efficiency of circ_0028934 shRNAs; B, CCK-8 proliferation assay in KYSE30 and KYSE150 cells after the knockdown of circ_0023984; C, EdU incorporation assay in KYSE30 and KYSE150 cells after the knockdown of circ_0023984; D, Colony formation assay in KYSE30 and KYSE150 cells after the knockdown of circ_0023984. E, Transwell migration assay in KYSE30 and KYSE150 cells after the knockdown of circ_0023984; F, Transwell invasion assay in KYSE30 and KYSE150 cells after the knockdown of circ_0023984. * P < 0.05; **P < 0.01; ***P < 0.001.

To verify the functional roles of circ_0023984 in ESCC cell growth, invasion, and migration, stable cell lines with circ-0023984 knockdown were established by lentiviral transduction of circ_0023984 shRNAs. RT-qPCR analysis showed that sh- circ_0023984#1 displayed the strongest silencing effect of circ_0023984 in KYSE30 and KYSE150 cells (Figure 2a), which was used for the subsequent experiments. CCK-8 proliferation assay demonstrated that the knockdown of circ_0023984 significantly suppressed the proliferation of ESCC cells (Figure 2b), which was further validated by the impaired ability to incorporate EdU for DNA synthesis (Figure 2c) and attenuated colony formation ability (Figure 2d). Circ_0023984 knockdown also suppressed the migration and invasion ability of KYSE30 and KYSE150 cells (Figure 2 e and f). Together, these data show that circ_0023984 is indispensable of the malignant cell phenotype of ESCC cells.

## Circ_0023984 negatively regulates miR-134-5p

**Figure 3.**
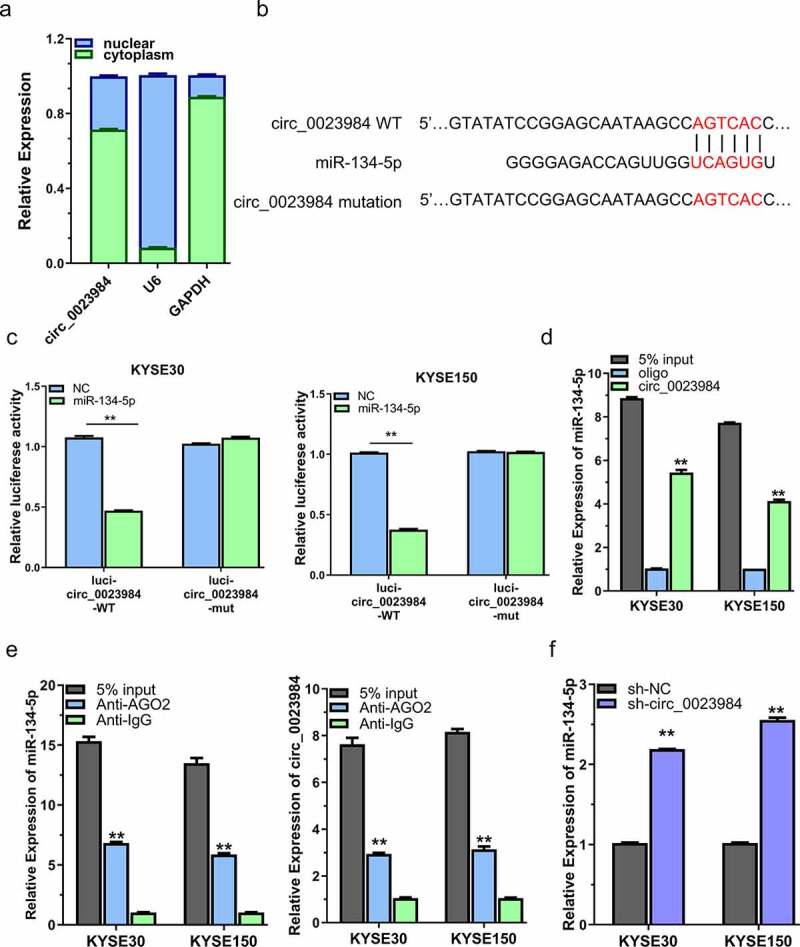
Circ_0023984 can target miR-134-5p.A, Subcellular fraction analysis to detect the cellular localization of circ_0023984 in KYSE150 and KYSE30 cells by RT-qPCR; B, circBank online tool predicts that circ_0023984 can target miR-134-5p; C, dual luciferase reporter assay in KYSE150 and KYSE30 cells using WT and MUT reporter, in the presence of miR-NC or miR-134-5p mimic; D, RNA pull-down assay using biotin-labeled circ_0023984 probe or control probe; E, RIP-qrt-PCR assay using anti-AGO2 antibody in KYSE30 and KYSE150 cells; F, RT-qPCR analysis of miR-134-5p level in KYSE30 and KYSE150 cells after circ_0023984 knockdown. * P < 0.05; **P < 0.01; ***P < 0.001.

To investigate the mechanism underlying circ_0023984, we first performed subcellular fraction assay to detect its cellular localization in KYSE150 and KYSE30 cells. RT-qPCR analysis revealed that circ_0023984 was predominantly localized in the cytoplasm (Figure 3a). We then used online bioinformatic tool circBank (http://www.circbank.cn/) to predict its target miRNA, and found that there was a binding site between circ_0023984 w miR-134-5p (Figure 3b). To validate this prediction, we applied dual-luciferase reporter assay using reporter containing WT or mutated (MUT) binding site. The presence of miR-134-5p mimic inhibited the luciferase activity in WT reporter but showed no effect in the MUT reporter or the NC in KYSE30 and KYSE150 cells (Figure 3c). Furthermore, the specific interaction between circ_0023984 with miR-134-5p was validated by RNA pull-down assay using biotin-labeled circ_0023984 probe, which showed that circ_0023984 was able to precipitate much more miR-134-5p as compared to control oligo probe (Figure 3d). In addition, anti Ago2 RIP-qrt-PCR assay showed that in KYSE150 and KYSE30 cells, in comparison with IgG group, the Ago2 antibody could enrich more circ_0023984 and miR-134-5p (Figure 3e). These data strongly suggest that circ_0023984 physically interacts with miR-134-5p. In cells with circ_0023984 knockdown, miR-134-5p level was remarkably increased (Figure 3f), indicating that circ_0023984 negatively regulates miR-134-5p expression.

## miR-134-5p interacts with CST4 mRNA

**Figure 4.**
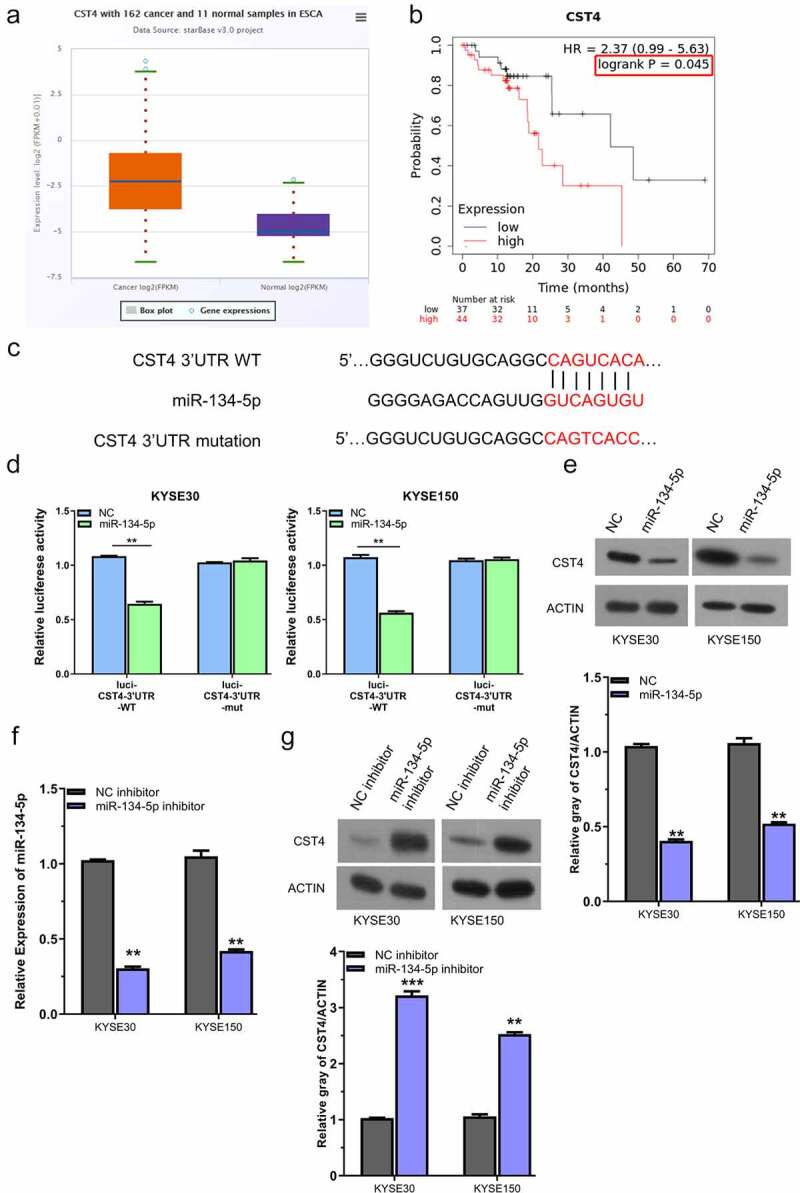
miR-134-5p negatively regulates CST4.A, CST4 showed high expression level in ESCC tissues (TCGA dataset with 162 ESCC samples and 11 normal samples); B, KM-plotter analysis of patients with high CST4 expression showed a poorer survival; C, Targetscan predicted that miR-134-5p targets the sequence of 2ʹURT in CST4 mRNA; D, dual luciferase reporter assay using WT and MUT reporter, in the presence of miR-NC or miR-134-5p mimic; E, WB analysis of CST4 protein level after miR-134-5p overexpressing in KYSE150 and KYSE30 cells; F, RT-qPCR analysis of miR-134-5p after the transfection with miR-134-5p inhibitor; G, WB analysis of CST4 level after the transfection with miR-134-5p inhibitor. * P < 0.05; **P < 0.01; ***P < 0.001.

The online tool starbase database (http://starbase.sysu.edu.cn/) revealed that CST4 was highly expressed in ESCC (TCGA dataset with 162 ESCC samples and 11 normal samples) (Figure 4a). As suggested by survival analysis, patients with high CST4 expression had a poorer overall survival (Figure 4b). Moreover, miR-134-5p could bind to the 3ʹUTR of CST4 mRNA as predicted by Targetscan online tool (Figure 4c), which was verified using dual-luciferase reporter assay in KYSE30 and KYSE150 cells (Figure 4d).

To confirm the regulatory role of miR-134-5p on CST4, we analyzed the protein level of CST4 in KYSE150 and KYSE30 cells after the transfection of miR-134-5p mimic, and found that miR-134-5p overexpression reduced CST4 protein level (Figure 4e). In contrast, when miR-134-5p level was reduced by the transfection of miR-134-5p inhibitor (figure 4f), the protein level of CST4 in KYSE150 and KYSE30 cells was elevated (Figure 4g). Therefore, we conclude that miR-134-5p interacts with CST4 mRNA and suppresses its expression.

## Circ_0023984 mediates the malignant phenotype of ESCC cells by targeting miR-134-5p/CST4 axis

**Figure 5.**
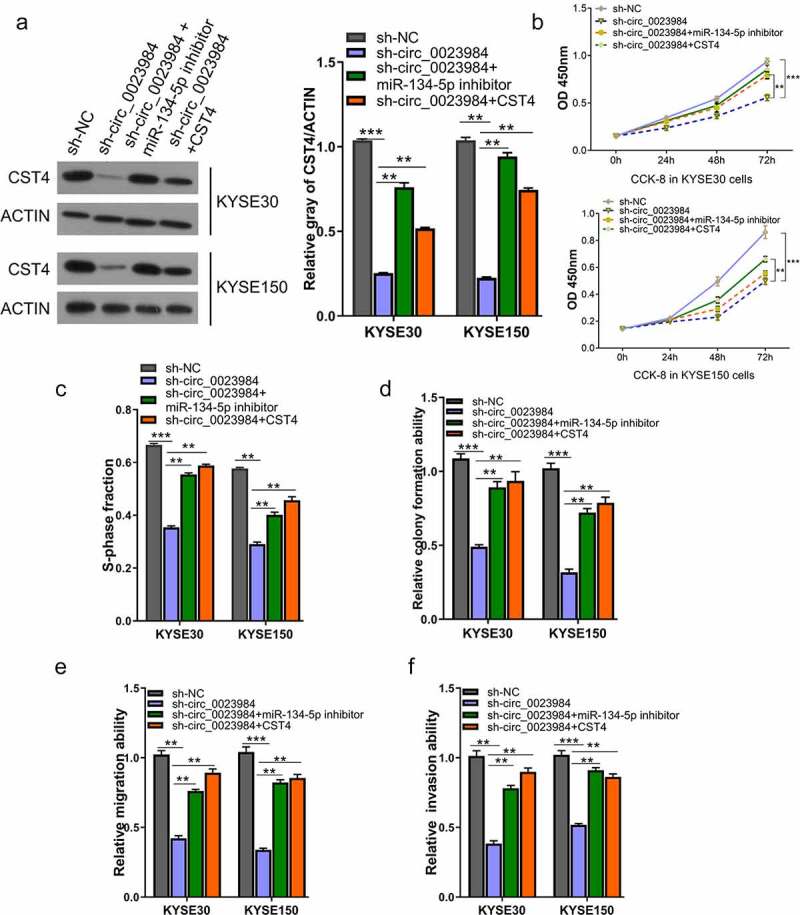
Circ_0023984 knockdown suppresses the malignant phenotype of ESCC by miR-134-5p/CST4 axis.A, WB analysis of CST4 protein levels in KYSE150 and KYSE30 cells with indicated treatments; B, CCK-8 proliferation assay at 0 h, 24 h, 48 h, and 72 h in different groups of KYSE30 and KYSE150 cells; C, EdU incorporation assay in different groups of KYSE150 and KYSE30 cells; D, Clonogenic assay in different groups of KYSE150 and KYSE30 cells; E, Transwell migration assay in different groups of KYSE150 and KYSE30 cells; F, Transwell invasion assay in different groups of KYSE30 and KYSE150 cells. * P < 0.05; **P < 0.01; ***P < 0.001.

To study whether miR-134-5p/CST4 axis mediates the effect of circ_0023984, ESCC cells with stable circ_0023984 knockdown was transfected with miR-134-5p or CST4 expression vector. Western bot showed that circ_0023984 knockdown reduced CST4 level, and miR-134-5p inhibitor or CST4 overexpression could rescue CST4 protein level upon circ_0023984 knockdown (Figure 5a). miR-134-5p inhibitor or CST4 overexpression also partially rescued the cell proliferation, DNA synthesis ability, as well as colony formation ability in KYSE150 and KYSE30 cells with circ_0023984 knockdown (Figure 5b-d). Transwell invasion and migration assay also revealed that miR-134-5p inhibitor or CST4 overexpression enhanced the migration and invasion ability of KYSE150 and KYSE30 cells with circ_0023984 knockdown (Figure 5e and 5f). Therefore, our data suggest that circ_0023984 regulates the malignant phenotype of ESCC cells by targeting miR-134-5p/CST4 axis.

## CST4 regulates effect of circ_0023984 on the *in vivo* tumorigenesis

**Figure 6.**
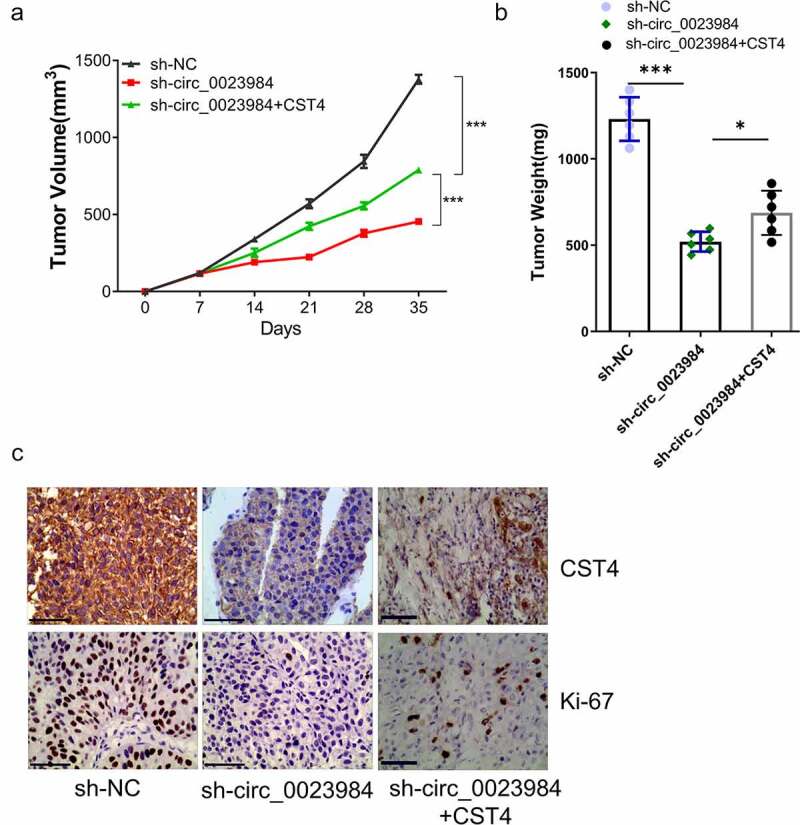
CST4 regulates effect of circ_0023984 on the in vivo tumorigenesis.The nude mice were subcutaneously injected with KYSE150 cells expressing sh-NC, sh-circ_0023984, sh-circ_0023984 and CST4 (n = 6 in each group). A, Subcutaneous xenograft volume in each group was measured; B, subcutaneous tumor weight of in each group was measured at the end of the experiment; C, immunohistochemistry (IHC) was performed to detect the expression of Ki-67 and CST4 in tumor sections of different groups. * P < 0.05; **P < 0.01; ***P < 0.001.

We also attempted to validate whether CST4 mediates the effect of circ_0023984 on the *in vivo* tumorigenesis in mouse model. We therefore generated the KYSE150 cells with stable circ_0023984 knockdown and CST4 overexpression (see methods). The nude mice were subcutaneously injected with KYSE150 cells expressing sh-NC, sh-circ_0023984, sh-circ_0023984 and CST4 (n = 6 in each group). We measured the size of subcutaneous xenograft of different groups every 7 days within 5 weeks. The results showed that the knockdown of circ_0023984 significantly inhibited subcutaneous tumorigenesis in nude mice, and the overexpression of SCT4 promoted tumorigenesis in cells with circ_0023984 knockdown (Figure 6a), which was also confirmed by the tumor weight measurement at the end of 5 weeks (Figure 6b). We also performed IHC staining of Ki-67 and CST4 in the xenograft tumor sections. In the tumors with circ_0023984 knockdown, the positive staining signals of Ki-67 and CST4 were largely reduced as compared to the tumor expressing sh-NC; while in tumors with circ_0023984 knockdown and CST4 overexpression, the level of Ki-67 and CST4 was partially increased (Figure 6c). These results suggest that CST4 mediates the effect of circ_0023984 on tumorigenesis in ESCC.

## Discussion

Patients with ESCC suffer from tumor progression and an overall poor prognosis [28]. Recently, numerous studies have demonstrated the critical roles of circRNAs in cancer genesis and development [29,30]. In ESCC, the deregulation of circRNAs has also been implicated in the malignant progression [31–33], which has been proposed as potential biomarkers and therapeutic targets.

The present work found that the elevated circ_0023984 expression in ESCC tissues in an ESCC dataset (GSE131969). The upregulation of circ_0023984 was also validated in clinical samples and cell lines. Interestingly, patients with high circ_0023984 expression were associated with a poorer overall survival, suggesting that circ_0023984 upregulation may contribute to the progression of ESCC. The functional roles of circ_0023984 in regulating the malignancy of ESCC cells were confirmed using circ_0023984 silencing experiments. Together, these data indicate that circ_0023984 is an oncogenic factor in ESCC cells and its high expression enhances the proliferation, migration and invasion of ESCC cells.

Noncoding RNAs such as circRNAs, lncRNAs, and pseudo-RNAs can act as competing endogenous RNAs (ceRNAs) to regulate gene expression by competitively binding to miRNAs [34]. CircRNAs have a closed-loop structure and are not easily degraded by exonucleases. CircRNAs that perform ceRNA functions are mainly derived from exons and localized in the cytoplasm, which act as ‘sponges’ by associating with AGO2 proteins and reply on their own MREs (miRNA reaction elements) to target miRNAs [35]. The adsorption of target miRNAs affects their ability to interact with downstream mRNAs, therefore regulating mRNA translation or stability [36,37]. In ESCC, circRNAs participate in the regulation of the malignant progression of tumor. For instance, circGSK3β promotes ESCC metastasis by enhancing β-catenin signaling [17]; circ_0006948 promotes ESCC progression via miR-490-3p/HMGA2 axis [31]; circNTRK2 promotes ESCC progression by upregulating NRIP1 expression via sponging miR-140-3p [32]; ciRS-7 adsorbs miR-876-5p and enhances the expression of MAGE-A to accelerate ESCC progression [38]. In a previous study, circ_0023984 was found to affect the progression of ESCC by sponging miR-433-3p, thereby regulating the expression of REV3L [16]. In our study, we further showed that circ_0023984 was mainly localized in the cytoplasm, and verified that it could be highly enriched by AGO2 antibody in RIP assay. Our data suggest that that circ_0023984 could act as ceRNA to sponge miRNAs in ESCC.

As there are several miRNA binding sites in circRNAs [6], our study identified and confirmed the interaction of circ_0023984 with miR-134-5p. miR-134-5p has implicated in the inhibition of cancer cell growth, invasion, and migration, such as in kidney cancer, colorectal cancer (CRC), and liver cancer [39–41]. However, its role in the biology of ESCC is unclear. Our data showed that miR-134-5p expression was increased in cells with circ_0023984 knockdown, and the effect of circ_0023984 silencing was partially rescued by miR-134-5p inhibitor. Our data indicate that circ_0023984 modulates ESCC cell growth, invasion and migration through sponging miR-134-5p.

miRNAs usually targets mRNA 3ʹUTR to regulate translation [42]. Indeed, we showed that miR-134-5p targets CST4 mRNA at its 3’ UTR and negatively regulates CST4 expression. CST4 is also called Cystatin-sa-III or salivary acidic protein 1, which is a secreted protein that inhibits protease hydrolysis reactions by binding to cysteine proteases [43]. Under normal conditions, CST4 is expressed in serum, saliva, tears, and seminal plasma at low levels. However, CST4 shows a high expression in gastrointestinal cancer cells and tissues [43]; and in gastric cancer study, CST4 upregulation contributes to the gastric carcinogenesis and progression by modulating NLFN2 signaling pathway [44]. In addition, CST4 can also remodel tumor microenvironment in ovarian cancer, and high CST4 expression is associated with the dismal survival of ovarian cancer patients [45]. In our study, we also showed that CST4 overexpression promotes the malignant phenotype in ESCC cells upon circ_0023984 knockdown. More importantly, CST4 overexpression enhances the tumorigenesis of ESCC cells with circ_0023984 knockdown in nude mice. Therefore, our data also support the oncogenic role of CST4 in ESCC cells.

## Conclusion

To sum up, this study demonstrated that the high circ_0023984 expression in ESCC contributes to its malignancy and tumorigenesis. We also showed that circ_0023984 modulated ESCC progression by targeting miR-134-5p/CST4 axis. Future studies are needed to elucidate the mechanism by which circ_0023984 is upregulated in ESCC.

## Data and material availability

The data is available from the corresponding author upon reasonable request.
